# Effectiveness of Cyanoacrylate in the Treatment of Dentin Hypersensitivity: A Systematic Review

**DOI:** 10.1155/2023/1465957

**Published:** 2023-08-23

**Authors:** Adriana da Silva Torres, Olga Beatriz Lopes Martins, Rejane Pereira Otoni, Kaio Henrique Soares, Marcelo Guimarães Torres, Parsa Firoozi, Olga Dumont Flecha

**Affiliations:** ^1^Dentistry Department, Faculty of Biological and Health Sciences, Universidade Federal dos Vales do Jequitinhonha e Mucuri, Rua da Glória n° 187, Centro, Diamantina, MG CEP 39100-000, Brazil; ^2^Student Research Committee, School of Dentistry, AJA University of Medical Sciences, Tehran, Iran

## Abstract

**Objective:**

To compare the effectiveness of cyanoacrylate to other treatments or placebo in the management of dentin hypersensitivity (DH).

**Materials and Methods:**

The present review was organized based on the preferred reporting items for systematic reviews and meta-analysis (PRISMA) guidelines. The search aimed to answer the following question: is cyanoacrylate effective in the treatment of DH when compared to other treatments or placebo? The following databases were used: PubMed/MEDLINE, Scopus, BVS, Web of Science, Cochrane, Clinicaltrials.gov, Portal Periódicos Capes, Google Scholar, and manual search. The evaluation process started with the information collected from the selected articles according to the Consolidated Standards of Reporting Trials (CONSORT).

**Results:**

Two randomized and five nonrandomized clinical trials were analyzed in the qualitative synthesis. The studies presented different cyanoacrylate formulations, different scales for evaluating pain, and different methods for provoking a painful stimulus. Cyanoacrylate-based products reduce DH in shorter follow-up periods and this reduction persisted throughout the study. The results varied according to the methods used to stimulate the pain. Only two articles showed a low risk of bias and a high level of scientific evidence.

**Conclusion:**

Although there is a limited number of studies in the scientific literature with appropriate methodological quality, the available evidence proves the effectiveness of cyanoacrylate in the treatment of DH. *Clinical Relevance*. Cyanoacrylate is easy to access, effective, easily applicable, and a low-cost product with satisfactory results.

## 1. Introduction

Dentin hypersensitivity (DH) is defined as a short and acute pain that appears from exposure to open dentin tubules [1]. It has been agreed that it is generated by chemical, thermal, tactile, or osmotic stimuli once it is not related to any other dental condition [2].

DH is one of the most common dental problems faced by dentists [3]. It could be caused by dental or periodontal damage as a result of enamel attrition and erosion, abrasion and abfraction, corrosion, periodontal tissue loss, or gingival recession [4–7]. Dental caries [8] and a fragile quantitatively defective enamel or hypomineralization [9] could also cause DH. In addition, other conditions could lead to dentin exposure and consequently unpleasant sensory experiences such as aggressive brushing, soft tissue dehiscence, and aging [7].

Several studies and theories have been proposed to explain how the stimuli could affect the dental pulp [10, 11]. Nowadays, it is widely accepted that the hydrodynamic theory could confirm the process involved in DH [12]. However, as different stimuli can affect the nerve fibers in different ways, researchers have contested how the hydrodynamic theory could explain all forms of DH. Evidence indicates that odontoblasts could play an important role in the mechanosensory mechanism associated with DH [13].

Although DH is a frequent dental issue, the prevalence presents a vast variation due to the different criteria to classify the same [14]. It is known that it could range from a minor inconvenience to the patient to a disturbance in the quality of life [15].

The wide number of treatment proposals is an obstacle to clinical professionals [14]. A large number of dental materials such as chemical components have been presented by pharmaceutical industries and recent research [16]. In this context, a few studies have tested the effectiveness of cyanoacrylate in reducing the symptoms presented in patients with DH [17, 18].

Cyanoacrylate adhesive is a commonly used material in medical sciences. Studies have shown its use in different areas of dentistry due to its properties [19]. From this evidence, this material has proved to be tolerated by human tissues, which could lead to the management of a large number of wounds in the oral cavity [20]. The role of cyanoacrylate in DH is related to its effective action in occluding the tubules preventing the displacement of fluids [18]. Thus, this agent could be a useful material to deal with DH as it is effective, easy to manipulate, and a low-cost product [15].

To verify how cyanoacrylate could be a viable therapy in the treatment of DH, this systematic review aimed to compare the effectiveness of cyanoacrylate to other treatments or placebo in the management of DH.

## 2. Materials and Methods

### 2.1. Protocol and Registration

The present review was organized based on the preferred reporting items for systematic reviews and meta-analysis (PRISMA) guidelines [21]. Furthermore, it was registered at PROSPERO with the protocol registration number: CRD42022370465.

### 2.2. Eligibility Criteria

This systematic review aimed to answer the following question: Is cyanoacrylate effective in the treatment of DH when compared to other treatments or placebo? The search was based on the following items of the PICOS question: population (any patient with DH not associated with pulpal and periodontal pathology), intervention (use of cyanoacrylate), comparator (any DH treatment or placebo), outcome (effectiveness of cyanoacrylate), and study design (randomized and nonrandomized clinical trials (RCTs)). Studies were excluded if they were observational studies, *in vitro*, animal studies, and review articles.

### 2.3. Search Strategy

All studies included in this review were obtained by an electronic search that took place in October 2022. The following databases were used: PubMed/MEDLINE, Scopus, BVS, Web of Science, Cochrane, Clinicaltrials.gov, Portal Periódicos Capes, Google Scholar, and manual search. No language limitation was considered to decrease the risk of language bias. All keywords were checked with the MeSH (Medical Subject Headings) database and then were used: (cyanoacrylate OR cyanoacrylates OR bucrylate OR enbucrilate) AND (dentin sensitivity OR dentin sensitivities OR dentine hypersensitivity OR dentin hypersensitivities OR dentin sensitivity OR dentin sensitivities OR tooth sensitivity OR tooth sensitivities OR DH OR dentin hypersensitivities). The search strategy has been presented in Table S1.

### 2.4. Study Selection

The articles underwent an independent and rigorous assessment by two reviewers (AST and OBLM) in two different stages. First, the reviewers independently selected all articles retrieved from the databases and manual search based on eligibility criteria after reading the titles and abstracts. Second, the full text of the selected papers was collected and appraised by the reviewers. Any disagreement about eligibility and any controversies between the two reviewers were resolved through a discussion to reach a consensus.

### 2.5. Data Extraction

The two authors extracted data from the selected studies and then double checked all the information. All the data obtained by the articles were tabulated with the following information: author and year of publication, country/region, study design, sample size, intervention, comparator, follow-ups, parameters of evaluation of hypersensitivity stimuli, and findings. The authors of the selected papers would be contacted at any time to provide insufficient data if necessary.

### 2.6. Risk of Bias

Two reviewers (MGT and KHS) independently evaluated the risk of bias. The evaluation process started with the information collected from the selected articles according to the Consolidated Standards of Reporting Trials (CONSORT). The data were tabulated following He et al. [22] and Belém et al. [23] with established criteria to qualify the methodology in different evidence levels. Studies were analyzed in five domains: sample size, randomization, allocation concealment, masking, and losses. The criterion was considered appropriate (A) when reported by the authors and explained. If it was only mentioned and not explained as B (partially reported). However, if it was not mentioned it was marked as C. Once all the criteria, or at least four, in the evaluated clinical trial were marked as A, it was rated as the level of evidence I. If it partially met the criteria (at most two evaluations C) it was rated as the level of evidence II and if it followed two criteria or less, it was rated as the level of evidence III (Table 1).

## 3. Results

**Figure 1:**
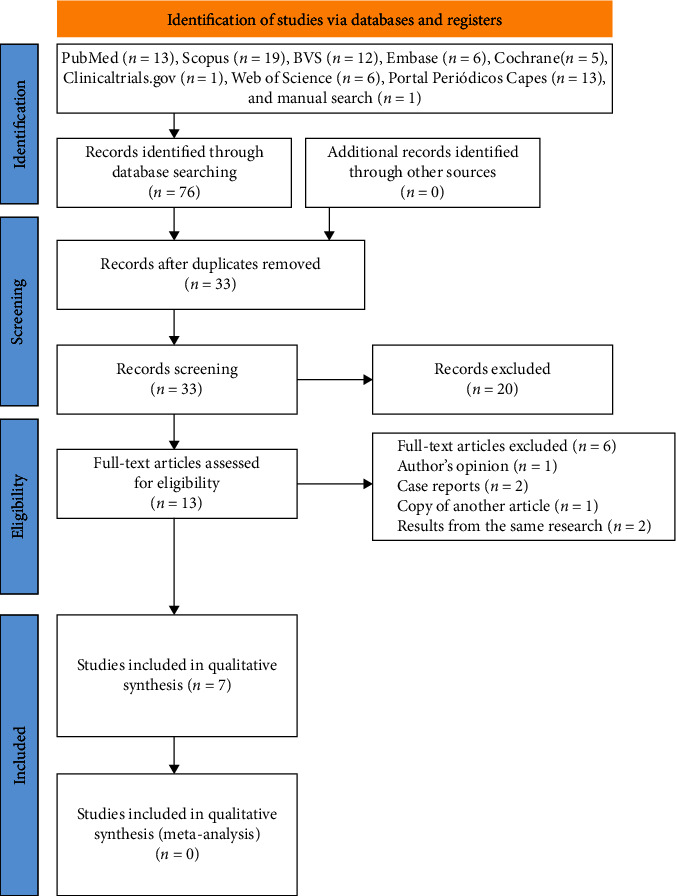
PRISMA flowchart showing the screening of the articles.

Initially, 76 papers were found in the databases. After removing duplicates, 33 papers were screened based on titles and abstracts. A total of 13 studies remained and were assessed for full-text evaluation. Hence, six papers and one specialization final paper were analyzed for qualitative synthesis (Figure 1). The characteristics of the included studies are presented in Table 2.

Two studies were RCTs and five were nonrandomized. Regarding the number of participants, it ranged from 11 [24] to 152 patients [17]. Only one study described how sample size calculation was performed [18].

Some studies used ethyl cyanoacrylate [18, 24–26], others N-butyl-2-cyanoacrylate, in the treatment of dental hypersensitivity [17, 27] and only one study did not refer to the brand or chemical composition of the cyanoacrylate used in the experimental group [28].

de la Caridad Perez-Alvarez et al. [17] did not compare the use of cyanoacrylate with another material. The other studies compared the efficacy of cyanoacrylate with low-level laser [18, 24], potassium oxalate 30% [27], Prime & Bonder [25], 33% sodium fluoride [28], and 5% fluoride varnish [26] for the treatment of DH.

The studies used different scales for pain assessment, such as Numerical Rating Scale (NRS) [18, 24], Visual Analog Scale [27], pain Level 1 and Level 2 [17], and discomfort levels from 0 to 3 [26, 28]. In addition, there were differences regarding the methods used to provoke the painful stimuli and different results for the different stimuli, such as cold air jet, Endo Ice, tactile stimulus, electrical stimulus, and mechanical stimulus.

It was found that cyanoacrylate-based products reduce DH in shorter follow-up times and this reduction persisted throughout the study and the results varied according to the methods used to the stimulate pain.

Only two articles in this review had a low risk of bias and a high level of scientific evidence [18, 24] (Table 1).

## 4. Discussion

Cyanoacrylate is a biocompatible compound that can block dentin tubules, which could be used in the treatment of DH [18, 28]. However, there is scarce evidence in the literature. This review found a few studies comparing the use of cyanoacrylate to other treatments, such as laser therapy, the use of adhesive systems, and compounds containing fluorine or potassium oxalate.

All evaluated studies have shown positive results regarding the use of cyanoacrylate in the treatment of DH. On the other hand, the papers that presented evidence level III must be interpreted with caution as the studies could present methodological bias [29].

RCT is considered the second level of evidence for clinical decisions and the gold-standard study design to evaluate the health interventions [29]. The low-methodological quality found in most of the studies could be explained by the fact that five papers [17, 25–28] are non-RCTs, which were evaluated with evidence level III. In RCTs, the randomization process could achieve results without bias among groups exposed to different forms of treatments. Also, to ensure that the results could not be affected by the participants' characteristics, the random assignment is performed in all groups [30].

In addition to randomization, other criteria are important to classify the levels of evidence in the present review. The sample size is important to determine the precise amount to obtain valid results [31]; the allocation concealment protects the assignment sequence until interventions, which prevents selection bias [32]; masking avoids subjectivities [33], determination bias and it controls the observer bias and protects the sequence after allocation [32]; finally, losses of participants could interfere in the initial equivalence in both experimental and control groups due to the absences [30].

Regarding the heterogeneity of the different interventions, all studies compared the effectiveness of cyanoacrylate for the treatment of DH [17, 18, 24–28]. All studies have shown satisfactory results regarding the use of cyanoacrylate in DH, although the response varied according to the method used to stimulate pain. The comparison of potassium oxalate 30% and cyanoacrylate showed better results for the oxalate after the 7th week of evaluating tactile stimuli and the absence of hypersensitivity until the end of the study [27]. When the results of electrical stimuli were evaluated, the difference was statistically significant until 6 weeks for both groups (experimental and control). The cyanoacrylate group showed a greater reduction in DH than potassium oxalate 30% [27].

The study comparing cyanoacrylate to laser therapy found a significant reduction in DH. The cyanoacrylate group showed a reduction in 24 hr (Endo Ice) and 120 days (air spray), while the laser therapy group showed its best efficacy in 120 days (Endo Ice) [24]. In the results presented by Flecha et al. [18], there was a statistically significant difference in the intergroup comparison only in 24 hr for the air spray and Endo Ice. In addition, it has been shown that during the other follow-ups, the air spray and Endo-Ice stimuli did not present any differences between the cyanoacrylate and laser therapy groups. It proves the noninferiority of cyanoacrylate in relation to the laser therapy [18].

The included studies used different follow-ups ranging from a total of 6 [17] to 180 days [18]. Also, it was found a significant reduction in pain immediately after the application of both potassium oxalate and cyanoacrylate [27] and 24 hr after the application of cyanoacrylate compared to 6 weeks of application of a paste of sodium fluoride 33% [28].

The studies performed reapplication of the interventional methods if the symptoms of DH remained [17, 27]. In the study of Flecha et al. [18, 24], authors applied three sessions at intervals of 48 hr for both laser and cyanoacrylate. Santos et al. [25] performed applications in a single session and four sessions. In the study conducted by Guimarães [26] three applications took place in three weeks. In these last two studies, significant reductions in DH after the reapplication of cyanoacrylate were observed. In the study by Javid [28], cyanoacrylate was reapplied after 6 weeks due to sensitivity caused by the erosion and abrasion.

The present review had notable strengths. First, this study conducted an electronic and manual search in the main available databases. Second, it followed a methodologic rigor to evaluate the level of evidence of the selected studies. All these points were important to ensure that the review has high-internal validity.

Some factors contributed to the limitations in this study such as the high heterogeneity in the interventions, low level of evidence of the selected studies, low number of available reports, different evaluation methods and follow-ups, and cyanoacrylate in different formulations. Therefore, it is difficult to compare the studies with different methodologies.

Cyanoacrylate is a useful compound that could be used in an emergency, it is easy to access, effective, easily applicable, and a low-cost product with satisfactory results [34]. Regarding these advantages, it is still necessary for more studies using this product in the treatment of DH, with a high-methodologic rigor to ensure a high level of evidence once there is not yet a gold standard treatment for DH [35, 36].

Recent systematic reviews concluded that the best therapy for DH seems to be an association of protocols with physical (laser) and chemical agents (neural agents and blockers), in addition to a detailed anamnesis and physical examination that allow an individualized treatment plan [36, 37]. Currently, therapies are capable of reducing DH in the short and medium term [37]. Several studies have been carried out using biomimetic hydroxyapatite. RCT studied the use of biomimetic hydroxyapatite in dentifrices and found favorable results in the reduction of hypersensitivity/pain values, superior to conventional dentifrices with fluoride [38]. Furthermore, an updated systematic review and meta-analysis concluded that the use of biomimetic hydroxyapatite in oral care products is a safer and more effective agent than fluoride in controlling dentin hypersensitivity and may be superior to other desensitizers [39]. Therefore, it is suggested that new RCTs compare cyanoacrylates with other desensitizing agents with methodological rigor and short, medium, and long-term follow-up times.

## 5. Conclusion

Although there are a few studies in the scientific literature of good methodological quality on the effectiveness of cyanoacrylate in the treatment of DH, all studies found that this material proves to be effective for this purpose.

## Figures and Tables

**Table 1:** Risk of bias assessment.

Author/Year/Country	Sample size	Randomization	Allocation concealment	Masking	Losses	EL
Flecha et al. [18, 24], Brazil	YES = A	YES = A	YES = A	YES = A	YES = A	I
Flecha et al. [18, 24], Brazil	NM = C	YES = A	YES = A	YES = A	YES = A	I
Naregal and Raju [27], India	NM = C	NM = C	NM = C	NM = C	YES = A	III
de la Caridad Perez-Alvarez et al. [17] Cuba	YES = A	NM = C	NM = C	NM = C	YES = A	III
Santos et al. [25] Brazil	NM = C	NM = C	NM = C	NM = C	YES = A	III
Guimarães [26] Brazil	NM = C	NM = C	NM = C	NM = C	YES = A	III
Javid et al. [28] Iran	NM = C	NM = C	NM = C	NM = C	YES = A	III

*Note*. Table template reference: Belém et al. [23]. Abbreviations: EL, evidence level; NM, not mentioned.

**Table 2:** Characteristics of selected studies.

Author/Year/Country/Study design	Sample size	Interventions/protocols	Evaluations/follow-up	Findings
Flecha et al., [18, 24]/Brazil/RCT, Double-blind, split-mouth	434 Teeth	TG - Super Bonder® CG - LLLT Protocol: 3x 48/48 hs	NRS: - Air spray = 4s - Tetrafluormethan spray (Endo Ice®) = 4s Baseline, 24 hr, 30, 90, and 180 days	Reduction in DH ≠ a significant. Within 24 hr better results were recorded for cyanoacrylate

Flecha et al., [18, 24]/Brazil/RCT, Double-blind, split-mouth -Pilot study	34 Hemiarch	TG - Super Bonder® CG - LLLT Protocol: 3x 48/48 hs	NRS: - Air spray = 5s - Tetrafluormethan spray (Endo Ice®) = 5s Baseline, 24 hr, 30, and 120 days	TG = Endo-Ice: 24 hr ≠ significant TG = Air blast: 120 days ≠ significant CG = Endo-Ice: 120 days ≠ significant

Naregal and Raju [27]/Índia/NRCT	45 Teeth 15 Patients	G1 (CG) = Distilled water G2 = Potassium oxalate 30% G3—N-butyl 2-cyanoacrylate Protocol: 1 min	VAS: - Tactile stimuli - Electrical stimulation Baseline, immediate and once a week for 12 weeks	Tactile stimulus: G2 and G3 = ≠ significant 7 weeks G2 = w/o DH until end Electrical stimulation: G2 and G3 = ≠ significant 6 weeks G3 = > DH reduction

de la Caridad Perez-Alvarez et al. [17]/Cuba/NRCT	152 Patients	- Chlorhexidine 0,2% = 1 min - Tisuacryl® = 60s	Pain evaluation: Level 1 = absence Level 2 = pain remains 2, 4, 5 e 6 days after intervention	Treatment with Tisuacryl: was successful in 96.7% of patients: Severe DH = 81.5% Light/moderate = 100%

Santos et al. [25]/Brazil/NRCT	655 Teeth 73 Patients	G1: Super Bonder® = 1 G2: Prime & Bond 2.1 = 1 G3: Super Bonder® = 4 G4: Prime & Bond 2.1 = 4	- Mechanical stimuli = exploratory probe n 5 - Thermal stimuli = air spray/Endo Ice® Baseline, weekly, and 60 days	Groups 3 and 4: statistically significant results in DH reduction

Guimarães [26]/Brazil/NRCT	60 Teeth 23 Patients	G1: Fluorniz® = 4 G2: Super Bonder® = 1	- Mechanical stimuli = exploratory probe - Thermal stimuli = water spray/air spray 7/7 days - 3 weeks	Super Bonder® Group: 93.3% - DH reduction Fluorniz® Group: 90% - DH reduction

Javid et al. [28]/Iran/NRCT	60 Patients	G1: NaF 33% = 6 G2: cyanoacrylate = 1	- Air spray for 1s 24 hr and weekly (6x)	Cyanoacrylate group: 25 = immediate and continuous relief. Fluoride Group: 21 = had lower scores than initial

*Note*. Table template font: Belém et al. [23]. Abbreviations: CG, control group; DH, dentin hypersensitivity; LLLT, low-level laser therapy; NaF, sodium fluoride; NRCT, nonrandomized clinical trial; NRS, Numerical Rating Scale; RCT, randomized clinical trial; TG, test group; VAS, Visual Analoge Scale.

## Data Availability

Data supporting this review article are available on request from the authors.

## Supplementary Materials

Supplementary Materials Table S1. Databases and search strategy.
