# Supplementary material for: Effectiveness of Cyanoacrylate in the Treatment of Dentin Hypersensitivity: A Systematic Review
Source: Int J Dent. 2023 Aug 23;2023:1465957. doi: 10.1155/2023/1465957 (PMC10469394; doi:10.1155/2023/1465957)
Supplement: Supplementary Materials — Table S1. Databases and search strategy. [file 1465957.f1.pdf]

BVS= 12 ARTICLES

(Cyanoacrylate OR Cyanoacrilates OR Bucrylate OR Enbucrilate OR Cianoacrilatos OR Embucrilato OR Bucrilato) AND (Dentin Sensitivity OR Dentin Sensitivities OR Dentine Hypersensitivity OR Dentine Hypersensitivities OR Dentine Sensitivity OR Dentine Sensitivities OR Tooth Sensitivity OR Tooth Sensitivities OR Dentin Hypersensitivity OR Dentin Hypersensitivities)=11

(Cyanoacrylate OR Cyanoacrilates OR Bucrylate OR Embucrilato OR Enbucrilato) AND (Dentin Sensitivity OR Dentin Sensitivities OR Dentine Hypersensitivity OR Dentine Sensitivity OR Dentine Sensitivities OR Tooth Sensitivity OR Tooth Sensitivities OR Dentin Hypersensitivity)=11+1

(Cianoacrilatos OR Embucrilato OR Bucrilato OR Enbucrilato) AND (Hipersensibilidade da dentina OR Sensibilidade dental OR Sensibilidade dentária)= 11

PUBMED= 13 ARTICLES

(Cyanoacrylate OR Cyanoacrilates OR Bucrylate OR Enbucrilate) AND (Dentin Sensitivity OR Dentin Sensitivities OR Dentine Hypersensitivity OR Dentine Hypersensitivities OR Dentine Sensitivity OR Dentine Sensitivities OR Tooth Sensitivity OR Tooth Sensitivities OR Dentin Hypersensitivity OR Dentin Hypersensitivities)= 13

GOOGLE SCHOLAR

Cianocrilatos AND Hipersensibilidade da dentina AND Terapêutica

(Cianocrilatos OR cianocrilato OR embucrilato) AND (sensibilidade dentinária OR Hipersensibilidade da dentina OR Sensibilidade dental) tratamento OR (terapêutica OR terapia)

CLINICAL TRIALS= 1 ARTICLE

Cyanoacrylate OR Cyanoacrilates OR Bucrylate OR Enbucrilate) AND (Dentin Sensitivity OR Dentin Sensitivities OR Dentine Hypersensitivity OR Dentine Hypersensitivities OR Dentine Sensitivity OR Dentine Sensitivities OR Tooth Sensitivity OR Tooth Sensitivities OR Dentin Hypersensitivity OR Dentin Hypersensitivities)= 1 (ARTIGO DA OLGA)

SCOPUS = 19 ARTICLES

(Cyanoacrylate OR Cyanoacrilates OR Bucrylate OR Enbucrilate) AND (Dentin Sensitivity OR Dentin Sensitivities OR Dentine Hypersensitivity OR Dentine Hypersensitivities OR Dentine Sensitivity OR Dentine Sensitivities OR Tooth Sensitivity OR Tooth Sensitivities OR Dentin Hypersensitivity OR Dentin Hypersensitivities)= 19 ARTIGOS

COCHRANE= 5 ARTICLES

(Cyanoacrylate OR Cyanoacrilates OR Bucrylate OR Enbucrilate) AND (Dentin Sensitivity OR Dentin Sensitivities OR Dentine Hypersensitivity OR Dentine Hypersensitivities OR Dentine Sensitivity OR Dentine Sensitivities OR Tooth Sensitivity OR Tooth Sensitivities OR Dentin Hypersensitivity OR Dentin Hypersensitivities)= 5

WEB OF SCIENCE= 6 ARTICLES

(Cyanoacrylate OR Cyanoacrilates OR Bucrylate OR Enbucrilate) AND (Dentin Sensitivity OR Dentin Sensitivities OR Dentine Hypersensitivity OR Dentine Hypersensitivities OR Dentine Sensitivity OR Dentine Sensitivities OR Tooth Sensitivity OR Tooth Sensitivities OR Dentin Hypersensitivity OR Dentin Hypersensitivities)= 6

PORTAL CAPES= 13 ARTICLES

(Cyanoacrylate OR Cyanoacrilates OR Bucrylate OR Enbucrilate OR Cianoacrilatos OR Embucrilato OR Bucrilato) AND (Dentin Sensitivity OR Dentin Sensitivities OR Dentine Hypersensitivity OR Dentine Hypersensitivities OR Dentine Sensitivity OR Dentine Sensitivities OR Tooth Sensitivity OR Tooth Sensitivities OR Dentin Hypersensitivity OR Dentin Hypersensitivities)=13
